# Development of the neurotrophic keratopathy questionnaire: qualitative research

**DOI:** 10.1186/s41687-020-00192-y

**Published:** 2020-05-04

**Authors:** Lindsey T. Murray, Julie McCormack, Ioana Grobeiu, Ingela Wiklund, Miriam Kimel, Floortje Van Nooten

**Affiliations:** 1 Evidera, 7101 Wisconsin Avenue, Suite 1400, Bethesda, MD 20814 USA; 2 Dompé farmaceutici S.p.A, Via Santa Lucia, 6, 20122 Milan, MI Italy; 3 Evidera, The Ark, 201 Talgarth Rd, London, W6 8BJ UK

**Keywords:** Patient-reported outcome, Instrument development, Interviews, Content validity

## Abstract

**Background:**

Neurotrophic keratopathy/keratitis (NK) is a rare disease of the cornea that can lead to anatomical loss of the eye. Little is known about the NK experience from the patients’ perspective. The objectives of this study were to examine the symptomatic experience and impacts of NK on patients and assess the overall comprehension, relevance, and content validity of a new questionnaire.

**Methods:**

This was a cross-sectional, qualitative study conducted with NK patients with varying levels of disease severity, recruited from one clinical site. One-on-one interviews using concept elicitation and cognitive interviewing techniques were conducted.

**Results:**

Fourteen NK patients participated; 64.3% were female (*n* = 9), mean age was 65.7 ± 13.3, and 14.3% (*n* = 2), 21.4% (*n* = 3), and 64.3% (n = 9) were classified as Mackie stage I, stage II, or stage III, respectively. Participants reported 24 concepts, including: redness (*n* = 12, 86%), sensitivity to light (*n* = 11, 79%), general discomfort (n = 9, 64%), dry eye (n = 9, 64%), reduced visual acuity (n = 9, 64%), blurred vision (*n* = 8, 57%), and eye fatigue (n = 8, 57%). No new concepts were reported after the 13th interview. The most frequently reported impacts included frustration (*n* = 10, 71%), driving impairment (n = 8, 57%), reading impairment (*n* = 7, 50%), difficulty watching television (n = 7, 50%), and concern with potentially losing their eyesight due to NK (*n* = 6, 43%). Participants provided positive feedback on the draft NK Questionnaire (NKQ) and felt that it was comprehensive and relevant to their experience with NK. Additionally, the recall period, instructions, item concepts, and response options were well-understood by participants. Minor revisions were made to the tool for consistency (i.e., the timeframe “in the past 7 days” was added to items 12–14); item 14 was modified to include “how often”; examples were added to item 9.

**Conclusions:**

The results of the concept elicitation portion of the qualitative study support the content validity of the draft NKQ. The clinically significant concepts identified in the literature and raised during concept elicitation are included as items in the questionnaire. Further assessment of the psychometric properties should be conducted in support of this new tool to measure the effect of new treatments on symptoms and impacts associated with NK.

## Background

Neurotrophic keratopathy (NK), also known as neurotrophic keratitis or neuroparalytic keratitis, is a rare disease of the cornea that is caused by lesions on the fifth (trigeminal) cranial nerve or its ophthalmic branch [1]. NK is characterized by a reduction (hypoesthesia) in or loss (anesthesia) of corneal sensitivity associated with poor corneal healing and can result in spontaneous epithelial breakdown, ulceration, infection, melting, and perforation of the cornea; it can ultimately lead to permanent loss of vision in the affected eye and anatomical loss of the eye [2]. Decreased stimulation of the tear gland is also possible due to damage to the trigeminal sensory fibers [1]. NK generally affects adults but can also affect children, and its estimated prevalence and incidence is less than 1.6/10,000 [3]. Typically, NK affects one eye, and can cause unilateral decrease of visual acuity in all stages.

The most common causes of loss of corneal sensitivity are herpes keratitis, chemical burns, long-term use of contact lenses, corneal surgery, [4] ablative procedures for trigeminal neuralgia, [5] acoustic neuroma surgery, and surgical procedures for reduction of jaw fractures [6]. Other less frequent causes are space-occupying intracranial masses (e.g., schwannoma, meningioma, and aneurysms), which can lead to compression of the nerve and reduce corneal sensitivity. Systemic diseases that may compromise trigeminal function are diabetes, multiple sclerosis, and leprosy [7]. NK may also be a complication of radiation therapy. Congenital causes, such as Ridley-Day syndrome, anhidrotic ectodermal dysplasia, Moebius syndrome, Goldenhar syndrome, and congenital corneal anesthesia, are very rare [8].

Diagnosis of NK is multi-faceted, including an assessment of systemic and ocular medical history, eye examination, and assessment of corneal sensitivity measured either qualitatively by touch or aesthesiometer [1, 9]. Patients frequently do not report symptoms related to NK, likely due to the reduced corneal sensitivity, which can create a discrepancy between clinical findings and patient report. Additionally, infective and immune keratitis should be evaluated as part of the differential diagnosis [1].

Available care regimens are palliative in nature, such as eye drops, lubricants, contact lenses, serum eye drops, amniotic membrane transplant, and tarsorrhaphy, and frequently address only one problem as they are intended only to protect the cornea in order to reduce the likelihood of disease progression [2]. Until recently, there were no approved treatments addressing the pathophysiology of the disease, the corneal nerves impairment. Recently, recombinant human nerve growth factor (rhNGF) administered topically demonstrated in two well-controlled randomized clinical trials to be safe and effective in achieving complete corneal healing in patients with stages 2 and 3 disease [10–12].

Clinical trials for eye treatments often include patient-reported outcomes (PROs) to capture symptomatic changes that only the patient can assess; however, there is no validated NK-specific PRO measure. Regulatory agencies, including the United States (US) Food and Drug Administration (FDA) and European Medicines Agency (EMA), have emphasized the importance of incorporating the patient voice into clinical trial outcomes using standardized, rigorously developed symptomatic measurement tools [13, 14]. In fact, the patient voice is becoming increasingly prominent in the drug approval process [15].

Establishing the content validity of a PRO in the population of interest (i.e., evidence that the instrument measures the concept of interest in NK) is the first step in developing a PRO that meets regulatory requirements for use in clinical trials to assess treatment benefit [13, 14]. Consistent with recommendations by the FDA’s road map to patient-focused outcome measurement in clinical trials [16] and the ISPOR PRO Content Validity Good Research Practices Task Force, [17, 18] a targeted literature review was conducted to identify key signs and symptoms of NK and eye diseases with similar symptomatology (dry eye, herpes keratitis, limbal stem-cell deficiency, and ocular burns). Additionally, a search was conducted to identify existing NK-specific PRO measures and/or instruments covering symptoms and signs of the disease. Searches were conducted in PubMed and Embase for 2007–2017. Additional searches in PROQOLID and clincialtrials.gov were conducted to identify PROs used in NK or other eye disease clinical trial programs. Symptoms and signs reported in the literature review related to NK included: redness, discomfort, inflammation, decreased corneal sensation, dryness, decreased tear production, reduced visual acuity, and irregular blinking (if bilateral disease) [1, 3, 19, 20]; however, some patients are asymptomatic [8, 19]. Although several existing PROs for eye disease were identified, [21–26] a gap analysis of each tool’s content showed that none of them adequately assessed the symptoms, signs, and impacts of patients with NK. Additionally, the development and validation samples did not include NK patients for any of the PROs evaluated; therefore, a new, draft NK-specific PRO measure was developed. In-line with the FDA Guidance on PRO development, concept elicitation interviews are typically conducted in patients with the disease of interest, followed by item generation and subsequent rounds of cognitive interviews to assess the content validity of the draft tool. However, given the rare nature of NK and the extremely limited number of patients available, a draft tool was developed prior to the patient interviews to maximize the feedback obtained from each individual patient. This methodology is in-line with the FDA’s recent draft guidance “Rare Diseases: Common Issues in Drug Development Guidance for Industry” and the ISPOR Rare Disease Clinical Outcomes Assessment Good Practices Task Force Report, [27, 28] which highlight the importance of utilizing multiple data sources, including the literature, to inform the natural history of disease and identify important symptoms common across patient subgroups. Both guidance documents suggest the application of a flexible and creative approach to overcoming sample size limitations and minimizing their impacts on PRO development.

The objectives of this study were to 1) examine the symptomatic experience and impacts of NK on patients and 2) document the development and content validity of a new, draft NK-specific PRO measure, in-line with FDA PRO Guidance requirements, adapted for development in a rare disease and suitable for inclusion in future clinical trials. Psychometric assessment of the tool to establish evidence for required measurement properties of reliability, validity, and sensitivity to changes in disease severity will be conducted as part of a future study.

## Methods

### Study design and procedures

This was a cross-sectional, qualitative study involving telephone sessions where participants were engaged in a one-on-one, semi-structured interview. No investigational drugs, devices, or invasive procedures were administered or evaluated as part of this study.

Participants with NK were recruited from a single clinical site located in Boston, MA, over a four-month period (December 2017–March 2018). The study was reviewed and approved by Western Institutional Review Board (WIRB), and all participants provided written informed consent. Inclusion criteria included age > 18; clinician-confirmed diagnosis of NK (mild, moderate, or severe) in one or both eyes, and measurable disease as defined by Mackie’s classification of: stage I (punctate keratopathy and/or corneal epithelial hyperplasia and irregularity), stage II (persistent epithelial defect [PED]), or stage III (corneal ulcer) [5]; able to fluently read, speak, and understand English; and willing and able to participate in an audio-recorded interview and complete questionnaires. A sub-set of three patients who had participated in a clinical trial for cenegermin (rhNGF) and had completed their last visit and exited the clinical trial within 12 months of enrollment in the current study were also recruited. Exclusion criteria included any uncontrolled active ocular infection (bacterial, viral, fungal, or protozoal) or uncontrolled active ocular inflammation not related to NK in the affected eye(s); new ocular diagnosis since the NK diagnosis; prior surgical procedure(s) for the treatment of NK (e.g., permanent tarsorrhaphy, conjunctival flap) with the exception of amniotic membrane transplantation or temporary tarsorrhaphy; or any other clinically relevant medical condition that, in the opinion of the investigator, would interfere with participating in an interview and/or completing the study procedures. Participants were remunerated a modest amount at completion of the study.

Two trained, experienced interviewers conducted the patient interviews using a standardized, semi-structured interview guide. Interviews were audio-recorded with the participant’s permission. The first section of the guide was open-ended and aimed to elicit key symptom and sign concepts and functional impacts from participants with minimal probing from the interviewer. Study participants were asked to describe their NK diagnosis history, symptoms, and functional impacts of their symptoms on daily life. Interviews were conducted such that participants had the opportunity to spontaneously describe and elaborate on their experiences. The interviewers ascertained words used to describe symptoms and functional impacts to understand the concepts that were most important to NK patients. Subsequent to this open-ended discussion of symptoms and functional impacts, Evidera interviewers probed on symptoms, signs, and impacts of NK mentioned in the literature that may not have been described previously by the patient.

Following the concept elicitation portion of the interview, study participants completed the draft Neurotrophic Keratopathy Questionnaire (NKQ) and were asked to provide feedback on the overall comprehension and clarity of the instructions, recall period, items, and response options.

### Assessments

#### Patient characteristics

Patients completed a brief sociodemographic form, which included questions about age, gender, race/ethnicity, living situation, employment status, marital status, education level, severity of NK, and overall health rating. Clinical characteristics of patients’ NK were captured in forms completed by study-site personnel.

#### Neurotrophic keratopathy questionnaire (NKQ)

The draft NKQ is a self-administered questionnaire that consists of 14 questions assessing the severity of 11 symptoms and signs and three impacts that may be associated with NK. Patients are asked to rate the severity of their NK-related symptoms and problems in the past 7 days. The initial draft of the NKQ was developed based on a review of literature and questionnaires assessing visual functioning and eye-related conditions, such as dry eye disease. Symptom item scores are rated on a five-point verbal rating scale, from “No, not at all” to “Very severe” for all items except item 5 (redness), which ranges from “No, not at all” to “A great deal”. Responses for the impact items (12–14) range from “Never” to “Always”. A preliminary conceptual framework is provided in Supplementary Fig. 1 and assumes that the 11 symptom and sign items will provide a NK severity score, with the three impact items providing supportive information on NK-specific quality-of-life.

#### National eye institute-visual functioning Questionaire-25 (NEI-VFQ-25)

The NEI-VFQ-25 is a validated instrument used to characterize participants in terms of vision-specific, health-related quality-of-life [22]. The NEI-VFQ-25 is a self-administered survey that consists of 25 vison-targeted questions in 11 domains covering general vision, difficulty with near- and distance-vision activities, difficulty with driving, vision-specific dependency, social functioning, role difficulties, limitation in peripheral and color vision, ocular pain, and mental health issues related to vision, plus an additional single-item question about general health [22]. All domains are scored on a 0–100 scale, ranging from 0 (worst) to 100 (best); higher scores indicate better vision-related function. Domain and composite scores were calculated in-line with developer guidelines [29].

### Analysis

Descriptive statistics (mean, standard deviation [SD], and frequency) were used to characterize the sample for sociodemographic and clinical characteristics, and to summarize data collected from the NEI-VFQ-25.

Transcripts from the interviews were analyzed with ATLAS.ti 7.5.18, [30] using a content analysis approach. A coding dictionary was developed based on the semi-structured interview guide to assist with the review. Participant quotes were grouped and summarized by thematic code, and saturation was evaluated for the concept elicitation data. The FDA has asked for evidence of “saturation” in qualitative research that is carried out in the development of PRO instruments [14]. Evidera used the definition of saturation presented in ISPOR PRO Good Practice Task Force, Part I [17] to establish and document saturation. Specifically, saturation is defined as the point at which no substantially new information/concepts continue to emerge beyond what has been mentioned in prior concept elicitation interviews. Saturation for the total sample was documented with a saturation grid, including tabulation of spontaneous and probed mentions of the symptoms. The exact number of participants needed to reach saturation cannot be exactly pre-determined since the number of participants needed to reach saturation depends on the complexity of the topic of interest and the diversity of experiences within the specific disease population of interest [31]. Additionally, concepts raised both spontaneously and after probing were compared with concepts raised in the literature for NK and related eye disease for the purpose of assessing whether concepts of importance were included in the draft tool. For the cognitive interview portion of the interview, analyses focused on assessing the clarity of instructions, interpretation of items, appropriateness of response options, and recall periods, and to ensure no items were missing from the tool. Due to the anticipated sample size limitations, only a single round of revision to the tool was planned subsequent to the completion of the concept elicitation/cognitive interviews.

## Results

### Sample description

Fourteen participants completed one-on-one interviews. Participants’ mean age was 65.7 ± 13.3 years (range: 42–82 years); the majority were female (*n* = 9, 64.3%) and white (*n* = 12, 85.7%) (Table 1). The mean time since date of diagnosis was 28.2 ± 17.4 months (range of 3–80 months), and participants were classified as Mackie stage I (*n* = 2, 14.3%), stage II (*n* = 3, 21.4%), or stage III (n = 9, 64.3%) (Table 2). For most participants, the location of the lesion was central (left eye: *n* = 5, 83.3%; right eye: *n* = 9, 90.0%), which is typical for NK. The underlying cause of NK varied across the sample (Table 3). The most frequently reported current treatments for NK were antibiotics (left eye: n = 3, 16.7%; right eye: n = 5, 27.8%), corticosteroids (left eye: n = 5, 27.8%; right eye: *n* = 6, 33.3%), and serum tears (left eye: *n* = 7, 33.3%; right eye: n = 5, 27.8%). The most frequently reported prior treatment for NK included antibiotics (left eye: n = 9, 40.9%; right eye: n = 7, 31.8%) and corticosteroids (left eye: n = 7, 31.8%; right eye: *n* = 11, 50.0%).

**Table 1:** Patient Characteristics

Characteristic	Total(***N*** = 14)
**Age (years)**	
Mean (SD)	65.7 (13.3)
Median [Range]	67.5 (42–82)
**Gender, n (%)**	
Male	5 (35.7%)
Female	9 (64.3%)
**Ethnicity, n (%)**	
Not Hispanic or Latino	14 (100.0%)
**Racial Background, n (%)**	
White	12 (85.7%)
Black or African American	1 (7.1%)
Other^a^	1 (7.1%)
**Current Living Situation, n (%)**	
Living alone	4 (28.6%)
Living with a spouse, partner, family, or friends	9 (64.3%)
Other^b^	1 (7.1%)
**Education Status, n (%)**	
Secondary school	2 (14.3%)
Associate degree, technical or trade school	1 (7.1%)
Some college	5 (35.7%)
College degree	2 (14.3%)
Postgraduate degree	4 (28.6%)
**Employment Status, n (%)**	
Full-time work	4 (28.6%)
Retired	8 (57.1%)
Disabled	2 (14.3%)
**Overall Severity of NK, n (%)**	
Moderate	5 (35.7%)
Severe	6 (42.9%)
Very severe	3 (21.4%)
**Overall Health, n (%)**	
Excellent	0 (0%)
Very good	0 (0%)
Good	10 (71.4%)
Fair	3 (21.4%)
Poor	1 (7.1%)
**NEI-VFQ-25, mean (SD) range**	
General Vision	54.3 (25.3), 0.0–80.0

^a^Other race includes: ‘White and Middle eastern’ (n = 1)

^b^Other living situation includes: ‘Long term skilled nursing care facility’ (*n* = 1)

Abbreviations: *NEI-VFQ-25* National Eye Institute-Visual Functioning Questionaire-25, *NK* Neurotrophic keratopathy, *SD* Standard deviation

**Table 2:** Clinical Characteristics

Characteristic	Total(N = 14)
**Date of Diagnosis (months)**	
Mean (SD)	28.2 (17.4)
Median [Range]	28.6 (3–80)
**Current Mackie Classification, n (%)**	
Stage I	2 (14.3%)
Stage II	3 (21.4%)
Stage III	9 (64.3%)
**Health Conditions, n (%)**^**a**^	
None	2 (14.3%)
Anxiety	2 (14.3%)
Arthritis	4 (28.6%)
Cancer	3 (21.4%)
COPD	2 (14.3%)
Depression	2 (14.3%)
Diabetes	1 (7.1%)
Hypertension	5 (35.7%)
Multiple sclerosis	1 (7.1%)
Psychosis	1 (7.1%)
Other^b^	6 (42.9%)

^a^Not mutually exclusive

^b^Other health conditions include: “asthma, stroke” (n = 1), “IBS, GERD, sleep problems” (n = 1), “LDL” (n = 1), “sarcoidosis” (n = 1), “hyperthyroidism, mitral valve regurgitation” (n = 1), and “mal de debarquement syndrome, CKD stage III, vertigo” (n = 1)

Abbreviations: *CKD* Chronic kidney disease, *COPD* Chronic obstructive pulmonary disease, *GERD* Gastroesophageal reflux disease, *IBS* Inflammatory bowel syndrome, *LDL* Low-density lipoprotein, *SD* Standard deviation

**Table 3:** Clinical Characteristics of Affected Eye

	Left Eye (N = 6)	Right Eye (N = 10)
**Affected Eye, n (%)**^**a**^	6 (100.0%)	10 (100.0%)
**Lesion Location, n (%)**		
Central	5 (83.3%)	9 (90.0%)
Peripheral	1 (16.7%)	1 (10.0%)
**Underlying Cause of NK, n (%)**		
Herpes keratitis	0 (0.0%)	1 (10.0%)
Herpes zoster keratitis	2 (33.3%)	2 (20.0%)
Corneal surgery	0 (0.0%)	1 (10.0%)
Dry eyes	0 (0.0%)	1 (10.0%)
Diabetes	0 (0.0%)	1 (10.0%)
Neurosurgical procedure	1 (16.7%)	0 (0.0%)
Other^b, c^	3 (50.0%)	4 (40.0%)
**Snellen Score, n (%)**		
20/20	1 (16.7%)	0 (0.0%)
20/150	1 (10.0%)	0 (0.0%)
20/1333	1 (10.0%)	0 (0.0%)
20/2666	2 (20.0%)	0 (0.0%)
20/8000	2 (20.0%)	2 (33.3%)
Light Perception	1 (10.0%)	1 (16.7%)

^a^Not mutually exclusive. Two participants had NK in both eyes

^b^Other underlying causes of NK in the right eye include: “partial limbal stem-cell deficiency” (n = 1), “herpes simplex keratitis” (n = 1), “Fuchs dystrophy” (n = 1), and “neurotrophic kertoconjunctivitis” (n = 1)

^c^Other underlying causes of NK in the left eye include: “partial limbal stem-cell deficiency” (n = 1), “herpes simplex keratitis” (n = 1), and “Stevens-Johnson syndrome” (n = 1)

Abbreviations: *NK* Neurotrophic keratopathy

The NEI-VFQ-25 was administered for descriptive purposes. Mean scores for each domain were: general health ratings = 42.9 (±18.2); general vision ratings = 54.3 (±25.3); ocular pain = 58.9 (±23.7); near activities = 49.4 (±30.9); distance activities = 53.0 (±23.0); social functioning = 74.1 (±22.7); mental health = 48.2 (±18.7); role difficulties = 43.8 (±24.9); dependency = 65.5 (±26.1); driving = 41.7 (±34.2); color visio*n* = 86.5 (±26.3); and peripheral vision = 50.0 (±24.0).

### Concept elicitation results

Results of the concept elicitation portion of the one-on-one interviews were documented in a saturation grid and demonstrated saturation of concepts. The most commonly reported NK symptoms and signs were redness (*n* = 12, 86%), sensitivity to light (*n* = 11, 79%), general discomfort (*n* = 9, 64%), dry eye (n = 9, 64%), reduced visual acuity (n = 9, 64%), blurred vision (n = 8, 57%), eye fatigue (n = 8, 57%), irritation or soreness (*n* = 7, 50%), and itching (n = 7, 50%). Of these, redness, eye fatigue, and itching were only reported when interviewers specifically probed on these symptoms. Participant descriptions were quantified mainly in terms of severity. Symptom frequency varied considerably, with participant reports ranging from “daily” to “once a month” to “always,” with variations based on situation (i.e., “only if it’s sunny outside”, “without a lens”). Among the total sample, 24 concepts were reported; 83% of these were reported in the first five interviews. Only three new concepts: decreased peripheral vision, cutaneous blistering of the eyelid, and irregular blinking were reported after the fifth interview. Concept saturation was reached after 13 interviews, indicating the sample size was sufficient.

The concepts reported by patients are largely consistent with findings from the literature and mapped well to items and response option structures included in the NKQ (Table 4). Sensitivity to light, blurred vision, and burning or stinging were not identified specifically as symptoms of NK in the literature, but were symptoms of related conditions and endorsed by patients during the concept elicitation portion of the interviews (*n* = 11, 79%, *n* = 8, 57%, and *n* = 5, 36%, respectively). Pain or discomfort and inflammation were reported in the literature for NK, but only endorsed by a small number of patients (*n* = 3, 21% and *n* = 4, 29%, respectively). Redness was also reported in the literature for NK, but was only reported upon probing by the interviewer. Decreased corneal sensation and tear production impacts were not reported by participants but are key clinical symptoms from the literature that are fundamental to NK diagnosis. Eye fatigue and itchiness were reported in the literature for related eye conditions and only endorsed upon probing.

**Table 4:** Concept Comparison across Literature and Patient Report

Concept	Reported in Literature: NK	Reported in Literature: Related Conditions^**a**^	Spontaneous	Probed	Total (N = 14) N, %	Item in NKQ
Redness	X	X	0	12 (86%)	12 (86%)	5
Sensitivity to light (photophobia)	NR	X	6 (43%)	5 (36%)	11 (79%)	7
General discomfort	X	X	2 (14%)	7 (50%)	9 (64%)	6
Dryness	X	X	3 (21%)	6 (43%)	9 (64%)	3
Reduction in visual acuity	X	X	6 (43%)	3 (21%)	9 (64%)	1
Blurred vision (or glare disability)	NR	X	4 (29%)	4 (29%)	8 (57%)	2
Burning or stinging	NR	X	0	5 (36%)	5 (36%)	4
Irregular blinking	X	X	0	1 (7%)	1 (7%)	8
Decreased corneal sensation^b^ (hypoesthesia or anesthesia)	X	X	NA	NA	NA	9
Increased or decreased tear production^c^	X	X	NA	NA	NA	10
Tearing (watery eyes)^c^	NR	X	NA	NA	NA	11
Pain or discomfort (acute, chronic, or prodromal)^d^	X	X	1 (7%)	2 (14%)	3 (21%)	None
Ocular fatigue^e^	NR	X	0	8 (57%)	8 (57%)	None
Irritation or soreness^e^	NR	X	2 (14%)	5 (36%)	7 (50%)	None
Itching (scratchiness)^e^	NR	X	0	7 (50%)	7 (50%)	None
Foreign body sensation (sand, grit in eye)^e^	NR	X	0	6 (43%)	6 (43%)	None
Inflammation (swelling of eye and/or eyelid)^e^	X	X	1 (7%)	3 (21%)	4 (29%)	None
Forced closing of eyelids^e^	NR	X	0	2 (14%)	2 (14%)	None
Discharge^e^	NR	X	1 (7%)	1 (7%)	2 (14%)	None
Headaches^e^	NR	NR	0	2 (14%)	2 (14%)	None
Sores or rash around the eyes^e^	NR	X	0	1 (7%)	1 (7%)	None
Cutaneous blistering^e^	NR	X	0	1 (7%)	1 (7%)	None
Punched-out oval or circular shape^e^	NR	X	0	0	0 (0%)	None
Sensitivity to cold^f^	NR	NR	1 (7%)	0	1 (7%)	None
Increased optic pressure^f^	NR	NR	1 (7%)	0	1 (7%)	None
Bleeding^f^	NR	NR	1 (7%)	0	1 (7%)	None
Decreased peripheral vision^f^	NR	NR	1 (7%)	0	1 (7%)	None
Floaters^f^	NR	NR	1 (7%)	0	1 (7%)	None

^a^Related conditions: dry eye, herpetic infections, and limbal stem-cell deficiency

^b^Decreased corneal sensation was not reported by participants; however, clinical confirmation of this symptom is required for NK diagnosis

^c^Tear production and watery eyes were not reported by participants; however, NK is caused by damage to the trigeminal nerve, which helps maintain the anatomical integrity of the ocular surface, including tear gland functioning, so assessment of this symptom is clinically important to NK

^d^Item 6 asks about discomfort but not pain

^e^Based on the review of the literature, several symptom probes were included in the interview guide to determine if the symptoms should be included in the questionnaire. For the most part, the symptoms were only reported by participants when probed, indicating these symptoms are of lower importance to patients

^f^These symptoms were not listed in the interview guide and were spontaneously reported by participants

Abbreviations: *NA* Not asked, *NK* Neurotrophic keratopathy, *NKQ* Neurotrophic Keratopathy Questionnaire, *NR* Not reported, *X* Reported

Descriptive comparisons were made between the NK severity groups (Mackie stages 1, 2, and 3) to see if there were any differences in reported symptoms and signs. All five participants who reported that they were blind in the eye affected with NK were classified as stage 3. Only the stage 3 participants reported experiencing pain in the eye affected with NK. No other differences in how participants described their symptoms were observed in this sample. Concept saturation was not assessed by sub-group, given the small sample sizes.

Participants also provided feedback on the impacts of NK on their daily activities. Most participants reported that NK interfered with their lives, with the most frequently reported impacts on their activities being driving impairment (*n* = 8, 57%), reading impairment (*n* = 7, 50%), and difficulty watching TV (n = 7, 50%). Participants also reported emotional impacts such as concerns with the possibility of losing their eyesight due to NK (*n* = 6, 43%) and frustration (*n* = 10, 71%).

### Cognitive interview results

Results of the cognitive interviews indicate that the questionnaire was comprehensive, and that the content (i.e., instructions, recall period, items, and response options) was clear and understandable. Participants demonstrated a good understanding of the instructions, indicating that no changes were necessary. Most participants (*n* = 9) also interpreted the recall period of the past 7 days correctly and reported that the 7-day period captured their NK-related symptoms. Three participants reported that 7 days is not long enough; however, no consistent suggestions for changes to the recall period were made and the suggestions that were provided exceed current regulatory guidelines for recall periods. The FDA has specifically stated a clear preference for daily recall, with justification for longer recall periods acceptable based on feedback such as what participants provided here (FDA guidance 2009). Therefore, no changes to the recall period were recommended. Overall, participants understood each of the items as intended. One participant commented that the items on burning and discomfort (items 4 and 6) were redundant, and another commented that the blurry vision and poor vision (items 1 and 2) were redundant. Furthermore, participants were able to define the response options appropriately in terms of increasing severity. Participants also reported that they could answer the question using each of the respective response options. Two participants commented that a non-applicable response option should be added, reporting that, for example, item 1 (poor vision), item 2 (blurry vision), and item 12 (worry about losing eyesight) would not apply to someone who has already lost their vision, and that some items need to be clarified as to whether the symptoms or problems occur with or without treatment; for example, participants commented that their responses to item 3 (dry eye) and item 10 (tear production) would vary depending on whether they were using eye drops. Two participants also commented that they did not see a distinction between the response options “a great deal” and “very much” in item 5. None of the suggestions were consistent enough to warrant changes to the tool, nor did participants express difficulty understanding the respective item concepts. Additionally, none of the participants indicated that there were missing concepts relevant to the NK symptomatic experience in the questionnaire.

No concepts that were raised during concept elicitation and supported by the literature were not already included in the draft NKQ item pool; thus, no items were added based on the results of the concept elicitation. Despite more than eight participants (57%) reporting eye fatigue and seven participants (50%) reporting itchiness as a symptom in the concept elicitation portion of the interview after prompting, none of the participants reported that these concepts were missing from the questionnaire during the cognitive debriefing and the concept was not identified as clinically significant to NK in the literature; therefore, items on eye fatigue and itchiness were not added to the NKQ. Likewise, decreased corneal sensation and tear production impacts were not reported by participants during concept elicitation, but were retained as items due to their clinical importance. Participant responses to each item were captured and showed preliminary evidence that the entire range of response options was used for each item (data not shown). Floor effects (> 20%) were observed for items 2–11 and ceiling effects (> 20%) were observed for items 1 and 2. Minor changes were made to items 12–14 for consistency with prior items (i.e., adding the timeframe “in the past 7 days” and modifying item 14 to ask “how often” participants have felt frustrated by their eyesight). Additionally, “can’t feel eye drops, or when the eye is being touched” were added to item 9 to enhance item comprehension. The current version of the draft NKQ after the concept elicitation and cognitive interviews consists of 11 symptom items and three impact items (Supplementary Fig. 1).

## Discussion

There has been increasing interest in incorporating the patient voice into clinical settings and trials using PRO data, [32–35] especially in rare diseases [36, 37]. The importance of including PRO data in obtaining labeling claims has been well established [15]. However, to obtain a symptomatic or health status label claim, it is imperative that the appropriate tool be implemented in the appropriate patient population (i.e., “fit for purpose”). This requires that sponsors identify, modify, or create tools that have sufficient content validity and psychometric robustness in the specific patient population to ensure reliable and responsive measurement of concepts important to patients that also have clinical value. These metrics can be particularly challenging in rare diseases. The ISPOR Clinical Outcome Assessment Emerging Good Practices Task Force released a report summarizing many of the challenges faced in identifying, selecting, developing, and implementing PRO assessments for use in rare disease clinical trials [27] and how these challenges apply to the FDA road map. Of relevance to the current study and the NK population are challenges to understanding the natural history of disease, heterogeneity of patient disease presentation and experience, measuring treatment benefit from the patients’ perspective, and lack of a disease-specific tool to use in that measurement. It was not unexpected to face some of these challenges in a population affected by NK, which is characterized by reduced corneal sensitivity and, in turn, lack of typical symptoms of corneal epithelial damage (i.e., intense or unbearable eye pain).

The results of the concept elicitation portion of this qualitative study underscore these challenges. Concepts raised by participants corroborated the symptoms and signs captured in the literature, but also expanded the list of symptoms and signs that NK patients associated with their disease beyond what has previously been reported. Additionally, two of the key clinical concepts raised in the literature (decreased corneal sensation and tear product impacts) were not reported by participants. There was also considerable variability among patients in terms of which symptoms and signs were experienced, not all of which could be attributed to differences in disease severity. Differences in the number of eyes affected and if vision had already been totally lost, as well as symptom and sign experience differences on- or off-treatment (i.e., lubricating eye drops reduced dry eye) also increase the complexity of the patient experience. This variation makes identifying the concepts to include in a disease-specific measure more challenging, as selected concepts should be broadly applicable to the population [27]. The goal for instrument development of a heterogenous disease must then be to begin with a broad range of potentially applicable items, acknowledging that some items may be removed subsequent to psychometric evaluation due to lack of sensitivity, overlapping concepts, or other poor performance metrics, such as reliability. The approach to scoring – developed following item and scale performance assessments – will also need to account for disease heterogeneity to truly reflect the patient experience and potentially may be more complex than approaches for disorders that present similarly across patients.

The draft NKQ is the first NK-specific PRO questionnaire measuring symptoms and signs developed in-line with the FDA PRO and Rare Disease Guidance [14, 28]. A targeted literature review of the symptoms, signs, and impacts of NK and related eye diseases was conducted as a first step. Existing PRO measures being used to evaluate symptoms, signs, and impacts of related eye diseases were also identified and evaluated for appropriate NK concept coverage and evidence of content and/or psychometric validity in an NK population. A gap analysis revealed that none of the existing measures were suitable for adaptation to NK; therefore, in order to develop a tool that is fit-for-purpose for regulatory claims, the draft NKQ was developed based on a broad range of applicable concepts from the literature. Concepts raised in the concept elicitation portion of this study mapped well to the draft NKQ, supporting its content validity. Additionally, participants provided positive feedback on the NKQ during the cognitive interview portion of each interview and felt that it was comprehensive and relevant to their experience with NK. The recall period, instructions, item concepts, and response options were well-understood by most participants. Furthermore, participants did not identify any missing symptomatic concepts, indicating that no important symptom concepts had been excluded from the tool. This was particularly important since more than half of the participants reported eye fatigue upon prompting during the concept elicitation portion, indicating that this was not a salient symptom to the patient’s NK experience and therefore an additional item on this concept was not added. A small number of participants identified potentially redundant items, and several items (redness, burning or stinging, irregular blinking, decreased corneal sensation, tear production, and watery eyes) were not associated with patients’ spontaneous report, indicating that these concepts may be of less importance to NK patients. Additionally, the item on irregular blinking was developed based primarily on the literature review, with only one participant reporting this symptom during item reduction. None of the participants reported that this item was irrelevant to them, and there was insufficient data from this small sample to conclude that any items should be removed prior to quantitative assessment.

Patients in this study described an array of impacts they experienced because of their NK disease, with frustration and worry or concern about losing eyesight being among the most commonly reported. The three impact items included in the draft NKQ (frustration, worry/concern about losing eyesight, and eyesight interfered with life) are designed to portray a broad overview of the impact experienced by NK patients and provide supplemental health-related quality-of-life data to guide interpretation of the symptomatic assessment items. The mean VFQ-25 general vision score is indicative of overall eye-related impacts. In this sample, the mean general vision score was 54.3, which is lower (i.e., worse) or similar to scores reported in three samples of patients with age-related macular degenerative (mean VFQ-25 general vision scores: 55.0–60.9, [38, 39]) and indicative of substantial quality-of-life impairment.

The main limitation of this study was the small sample size, which is reflective of a rare disease, but it did not allow for an iterative approach to instrument development with concept elicitation interviews followed by cognitive interviews. Also, all participants were recruited from a single clinical site and were predominantly female and white, which may limit the generalizability of the sample. However, patients with a range of NK severities were included in this study, ensuring as diverse a clinical sample as possible was included and concepts impacting the full range of disease severity were captured. Additionally, recruitment through a single study is common in rare diseases where health services are frequently limited to just one or two medical centers nationally or even internationally.

Concept saturation is defined as the point at which additional sampling provides no new information and serves no functional purpose [31]. In this study, additional interviews may have provided increased confidence in content validity beyond a single patient not reporting any new concepts; however, given the rare nature of the disease, this was simply not possible and the a priori concept saturation threshold was achieved with the 14 interviews, with 83% of concepts raised in the first five interviews. Several patients were also blind in the affected eye, which limited the applicability of some of the draft NKQ items to their current experience. Additionally, due to the length of the interviews, the opportunity to ascertain from the patient perspective what assessment timepoints during a potential clinical trial might be or what changes in the scores could be meaningful was not possible.

The draft NKQ items were based on the literature review of NK-specific symptoms, as well as symptoms identified in related eye diseases. The literature review primarily identified clinical diagnosis and management articles aimed at healthcare providers. Despite a substantial overlap, the concepts identified from the literature vs. by participants did not align perfectly, likely due to differences in the perception of disease-specific, clinically meaningful signs and symptoms between clinicians and patients. However, all concepts identified during the concept elicitation portion of each interview and supported by the NK-specific literature were already included in the draft NKQ item pool, assuring that the draft NKQ fully captures all NK-relevant concepts. Additionally, as described in Patrick et al. (2011) [18], no established thresholds exist for making decisions to modify a draft tool based on data collected from cognitive interviews. Instrument developers must determine what changes are required based on clear issues with concept clarity, recall considerations, and recommended modifications. In this study, in cases where only one or two participants reported an issue with item comprehension or made a suggestion to change the tool due to perceived item redundancy, it was deemed insufficient evidence to move forward with revisions to the tool. Item-level analysis indicated that a number of items may be eligible for deletion based on floor and/or ceiling effects; however, no conclusions could be drawn based on this preliminary sample. A conservative approach to instrument development was utilized in this study and no items were removed from the item pool after cognitive interviews. Further testing of the NKQ should be conducted in a future clinical trial of NK. Data from this study could be utilized to examine item performance characteristics, identify items for reduction, develop an appropriate scoring algorithm, and subsequently assess the reliability, validity, responsiveness, and responder thresholds of the NKQ.

The present qualitative study had several notable strengths. Participating patients were generally representative of patients expected to be recruited in future NK clinical trials. The draft NKQ was developed in-line with the FDA PRO and Rare Disease guidance documents and maximized patient input from a rare disease population by developing a draft tool grounded in the literature ahead of concept elicitation interviews. Interviews were then split into a concept elicitation section to ensure concept saturation was achieved. Reflective of the modified methodology in a rare disease population, analysis of the concept elicitation section provided evidence that concepts raised by participants mapped to the draft NKQ items and no clinically important concepts were missing from the draft tool. The second part of each interview focused on cognitively assessing the draft NKQ and ensuring the recall period, instructions, item concepts, and response options were understood and relevant to participants. Justified flexibility in rare disease population regulatory submissions, including PRO development, is understood [27] and has been supported by regulatory agencies historically [40].

## Conclusion

This study represents the first step in the development of the NKQ. The evidence gained in this qualitative study provides support for the content of the draft NKQ among adult patients with NK. The concepts included in the draft NKQ are important to patients with NK; the items, response options, and recall period are understood and acceptable, supporting establishment of the NKQ’s content validity. Additional quantitative work to evaluate item characteristics, item reduction, scoring, and assessment of the reliability, validity, responsiveness, and responder thresholds is required to provide further evidence that the NKQ is fit-for-purpose in regulatory submissions and demonstrate its ability to measure the effect of new treatments on symptoms and impacts associated with NK.

## Supplementary information

**Additional file 1:Figure S1.** Conceptual Framework of the Draft NKQ.

## Data Availability

The data from this study is not publicly available due to concerns regarding participant confidentiality.

## Abbreviations

EMA: European Medicines Agency
FDA: Food and Drug Administration
NEI-VFQ-25: National Eye Institute-Visual Functioning Questionaire-25
NK: Neurotrophic keratopathy/keratitis
NKQ: Neurotrophic Keratopathy Questionnaire
PED: Persistent epithelial defect
PRO: Patient-reported outcome
rhNGF: Recombinant human nerve growth factor
SD: Standard deviation
US: United States
WIRB: Western Institutional Review Board
