# Supplementary material for: Development of the neurotrophic keratopathy questionnaire: qualitative research
Source: J Patient Rep Outcomes. 2020 May 4;4:30. doi: 10.1186/s41687-020-00192-y (PMC7198680; doi:10.1186/s41687-020-00192-y)
Supplement: Supplementary file 1 — Additional file 1:Figure S1. Conceptual Framework of the Draft NKQ. [file 41687_2020_192_MOESM1_ESM.pdf]

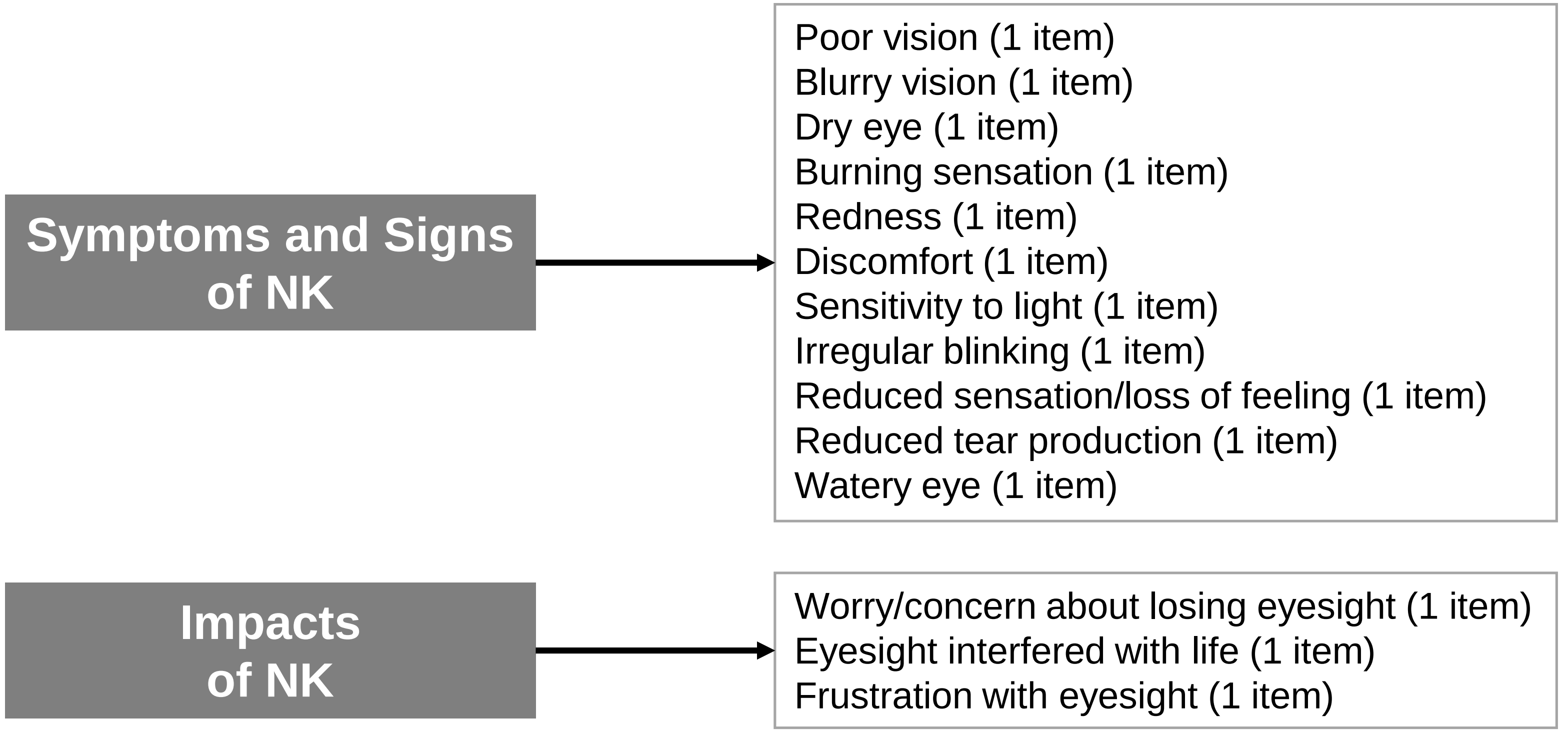
Symptoms and Signs of NK
Poor vision (1 item)
Blurry vision (1 item)
Dry eye (1 item)
Burning sensation (1 item)
Redness (1 item)
Discomfort (1 item)
Sensitivity to light (1 item)
Irregular blinking (1 item)
Reduced sensation/loss of feeling (1 item)
Reduced tear production (1 item)
Watery eye (1 item)
Impacts of NK
Worry/concern about losing eyesight (1 item)
Eyesight interfered with life (1 item)
Frustration with eyesight (1 item)
